# Hemodynamics and extracardiac causes of cardiac tamponade: a diagnostic and therapeutic dilemma

**DOI:** 10.1093/qjmed/hcag056

**Published:** 2026-02-20

**Authors:** Shan Zaidi, Nikita Jhawar, Vivek Kulkarni

**Affiliations:** Cooper Medical School of Rowan University, Camden, NJ, USA; Department of Cardiovascular Disease, Cooper University Hospital, Camden, NJ, USA; Cooper Medical School of Rowan University, Camden, NJ, USA; Department of Cardiovascular Disease, Cooper University Hospital, Camden, NJ, USA

Learning point for clinicians This case highlights the importance of recognizing extracardiac factors that may cause cardiac tamponade and warrant a tailored diagnostic and therapeutic approach. Judicious use of interventions is important in alleviating clinical and echocardiographic tamponade.

## Background

Cardiac tamponade is typically attributed to pericardial fluid accumulation; however, extracardiac compression from large pleural effusions can similarly impair cardiac filling and produce tamponade physiology. Although pleural effusions are relatively common, especially in postoperative cardiac patients, their potential to cause tamponade is rarely recognized. In this case, a significant pleural effusion, despite only a small pericardial effusion, resulted in tamponade physiology and resolved following thoracentesis.

## Case presentation

One month prior to presentation, a 68-year-old woman underwent a bioprosthetic mitral valve replacement for severe mitral regurgitation. Postoperatively, she developed a localized anterior pericardial effusion with early tamponade physiology on transthoracic echocardiogram (TTE) without clinical tamponade. The patient was observed and discharged to a subacute rehabilitation facility as she was asymptomatic and hemodynamically stable. During the 2 weeks preceding presentation to the emergency department, she developed hypotension and progressive generalized fatigue.

On arrival, blood pressure was 108/51 mmHg, pulse 104 beats/min, respiratory rate 16 breaths/min and oxygen saturation was 97%. Physical examination notably revealed weakened radial pulses with inspiration and a decrease in systolic blood pressure by approximately 13 mmHg during inspiration, consistent with pulsus paradoxus. Lung sounds were diminished only on the right side. The patient denied chest pain, palpitations or lightheadedness. Electrocardiogram demonstrated normal sinus rhythm with occasional premature ventricular contractions and low voltage.

**Figure 1:**
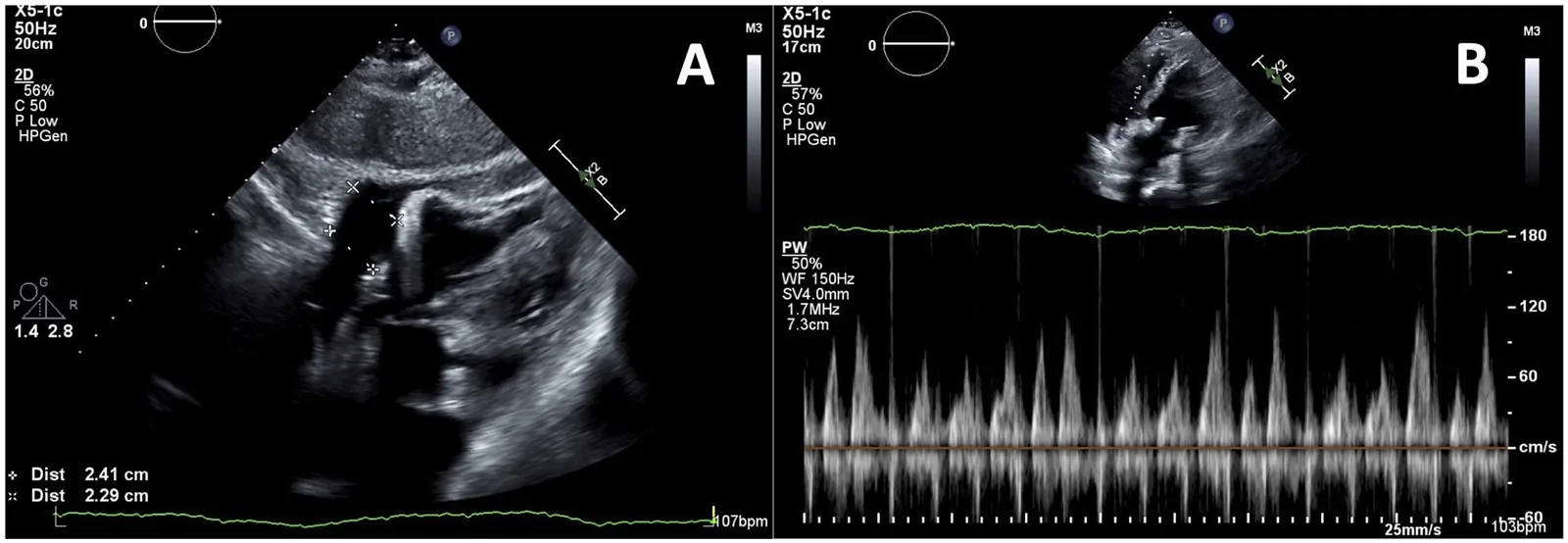
TTE demonstrating a focal pericardial effusion adjacent to the right ventricle’s free wall (**A**). Pulsed-wave Doppler of the mitral valve inflow demonstrating marked respiratory variation (**B**).

Subsequent TTE showed mid to apical hypokinesis of the left ventricle with an estimated left ventricular ejection fraction (LVEF) of 30–35%. The focal anterior pericardial effusion containing hematoma was corroborated on TTE (Figure 1A). There was a localized, partially echodense material inferolateral to the basal to mid right ventricle free wall and right atrium. Respiratory variation was seen in tricuspid and mitral inflow views TTE (Figure 1B). The inferior vena cava (IVC) had a dilated diameter with reduced inspiratory collapse.

By day 2 of admission, physical examination revealed elevated jugular venous pressure (14 mmHg) and a positive Kussmaul sign. Breath sounds were decreased at the lung bases, more prominent on the right. By day 4 of admission, she endorsed significant dyspnea with exertion. On day 6, repeat TTE showed a persistent moderate, loculated pericardial effusion adjacent to the right atrium and right ventricle. LVEF was stable at an estimated 30–35% and the IVC was newly dilated with reduced inspiratory collapse leading to increased concern for tamponade. Repeat computed tomography chest on day 6 showed that the right pleural effusion increased in size, with the hemopericardium relatively unchanged in size compared to the computed tomography angiogram on admission.

Given the simultaneous presence of a large pleural effusion, bedside thoracentesis was performed first prior to pericardial window and drained 1.8 l of fluid. The patient’s symptoms improved dramatically after thoracentesis. Post procedural TTE showed a persistent moderate-sized, focal pericardial effusion unchanged from prior, but now with normal IVC size and no respiratory variation, suggesting resolution of cardiac tamponade.

## Discussion

Although less common than primary pericardial sources, extracardiac etiologies of tamponade represent an important cause of hemodynamic compromise. Compression from the right-sided pleural effusion on the heart and great vessels, and the subsequent impaired venous return, impaired cardiac filling, likely resulted in the tamponade physiology observed.^1–3^ This premise was supported by resolution of tamponade after thoracentesis, which reiterates the importance of judicious therapeutic interventions that prioritize patient safety and high value care.^3^ We demonstrate removal of the pleural fluid decreased the intrathoracic pressure, which improved venous return, left ventricular preload, stroke volume and thus cardiac output.^3–5^ This was evidenced by IVC collapsibility after thoracentesis due to reduction in intrathoracic pressure, suggesting an improvement in pericardial filling.^6^ Recognizing extracardiac tamponade is crucial to optimally guiding procedural management.

## Ethics approval

Written patient consent was obtained for the described case.

## References

[1] Kearns MJ , Walley KR. Tamponade. Chest 2018; 153:1266–75. 10.1016/j.chest.2017.11.003 29137910

[2] Adler Y , Ristić AD, Imazio M, Brucato A, Pankuweit S, Burazor I, et al Cardiac tamponade. Nat Rev Dis Primers 2023; 9:36. 10.1038/s41572-023-00446-1 37474539

[3] Kopterides P , Lignos M, Papanikolaou S, Papadomichelakis E, Mentzelopoulos S, Armaganidis A, et al Pleural effusion causing cardiac tamponade: report of two cases and review of the literature. Heart Lung 2006; 35:66–7. 10.1016/j.hrtlng.2005.07.001 16426937

[4] Alam HB , Levitt A, Molyneaux R, Davidson P, Sample GA. Can pleural effusions cause cardiac tamponade? Chest 1999; 116:1820–2. 10.1378/chest.116.6.1820 10593815

[5] Hermansen JF , Juhl-Olsen P, Frederiksen CA, Christiansen LK, Hørlyck A, Sloth E. Drainage of large pleural effusions increases left ventricular preload. J Cardiothorac Vasc Anesth 2014; 28:885–9. 10.1053/j.jvca.2013.11.018 24656616

[6] Porter TR , Shillcutt SK, Adams MS, Desjardins G, Glas KE, Olson JJ, et al Guidelines for the use of echocardiography as a monitor for therapeutic intervention in adults: a report from the American Society of Echocardiography. J Am Soc Echocardiogr 2015; 28:40–56. 10.1016/j.echo.2014.09.009 25559474

